# New Application of the Inhalation of Chloroform

**Published:** 1851-10

**Authors:** 


*New Application of the Inhalation of Chloroform.*—M. Guisard, doctor of medicine, and member of the French Assembly, has written to *L'Union Médicale*, to state that his son, about three years of age, and affected with phimosis, had just been relieved from very distressing symptoms by inhalations of chloroform. It appears that the little patient experienced great agony in passing urine, and the distress was so great that he overcame the necessity of performing this function, and obstinately refused to relieve his bladder.

He was placed under the influence of chloroform, so that the phimosed prepuce could be incised, and hardly had its effects taken place than the urine began to flow, and the bladder was emptied. The next day, the child was equally obstreperous, and the same means brought about the same results. M. Guisard thinks that these facts may open a new path for the treatment of the retention of urine or of fæcal matter.—*London Lancet.*

